# Effects of dietary dried distillers’ grains with solubles and NSP enzyme supplementation on growth performance, intestinal morphology, immunity, and economic efficiency in broilers

**DOI:** 10.3389/fvets.2025.1752220

**Published:** 2026-01-26

**Authors:** Amr M. Shams-Eldin, Ahmed Sayed-Ahmed, Ahmed R. Elbestawy, Moustafa Elhamouly, Fuad Saleh, Whad Fayed, Hamada A. Ahmed

**Affiliations:** 1 Department of Nutrition and Clinical Nutrition, Faculty of Veterinary Medicine, Menoufia University, Shebeen El-Kom, Egypt; 2 Department of Anatomy and Embryology, Faculty of Veterinary Medicine, Menoufia University, Shebeen El-Kom, Egypt; 3 Department of Bird and Rabbit Diseases, Faculty of Veterinary Medicine, Menoufia University, Shebeen El-Kom, Egypt; 4 Department of Cell Biology and Histology, Faculty of Veterinary Medicine, University of Sadat City, El Sadat City, Egypt; 5 Devenish Nutrition Ltd., Belfast, Ireland; 6 Department of Nutrition and Clinical Nutrition, Faculty of Veterinary Medicine, Damanhour University, Damanhour, Egypt

**Keywords:** broilers, DDGS, growth performance, immunity, intestinal morphology, NSP enzymes

## Abstract

**Introduction:**

This study evaluated the effects of incorporating varying levels of dried distillers’ grains with solubles (DDGS) and non-starch polysaccharide (NSP) enzymes into broiler diets to assess their impact on growth performance, immune function, intestinal histomorphology, and economic efficiency.

**Methods:**

A total of 240 one-day-old broiler chicks (Avian 48; initial weight 51.57 ± 4.6 g) were randomly assigned to eight dietary treatments (30 birds each). The treatments consisted of four DDGS inclusion levels (0, 5, 10%, or 20%) provided either with or without NSP enzyme supplementation.

**Results:**

Growth performance indicated that moderate DDGS inclusion (5–10%) with enzymes improved body weight gain and feed conversion ratio during the starter and grower phases, whereas 20% DDGS without enzymes led to poor performance. While most serum biochemical parameters remained unaffected (*p* > 0.05), triglycerides and globulin increased significantly (*p* < 0.05) in enzyme-supplemented groups. Furthermore, enzyme-supplemented groups showed significantly higher IgA and IgM levels, alongside higher white blood cell counts and H/L ratios at 5% and 10% DDGS levels. Histological examination demonstrated improved villus height and crypt structure in enzyme-supplemented diets, while 20% DDGS without enzymes negatively affected gut morphology.

**Discussion/Conclusion:**

Economically, the 5% DDGS diet with enzymes achieved the lowest cost and highest net profit, whereas the 20% DDGS group without enzymes recorded the poorest economic return. In conclusion, incorporating DDGS at 5–10% with enzyme supplementation enhances growth performance, immune stimulation, and intestinal health in broilers without compromising physiological functions.

## 1 Introduction

The poultry industry constantly seeks cost-effective and sustainable feed ingredients to enhance profitability while ensuring optimal bird performance. Dried distillers’ grains with solubles (DDGS), a byproduct of ethanol production, has emerged as a valuable alternative protein and energy source in broiler diets due to its high nutritional content, wide availability, and potential to lower feed costs. Moreover, DDGS has been recognized as a functional feed additive that can enhance poultry productivity through microbiota-mediated mechanisms, providing further support for its use in sustainable poultry production (1). Global biofuel production is expected to increase by over 20% between 2022 and 2027, emphasizing the rising significance of DDGS as a co-product (2). However, the inclusion of DDGS in poultry diets is often restricted due to its high levels of non-starch polysaccharides (NSPs). Corn DDGS contains approximately 26.5% total NSPs, including 3.55% soluble and 23.5% insoluble fractions, along with 21.5% arabinoxylans and 0.32% β-glucans (3). These components can negatively impact growth performance, gut health, and nutrient absorption efficiency.

Previous research indicated that moderate levels of DDGS can support broiler growth when properly balanced within the diet. Young broilers, in particular, are sensitive to feed quality because their digestive system are not fully developed until approximately 2 weeks of age (4). Water-soluble NSPs, when included in young chick diets, have been shown to reduce nutrient digestion and absorption by increasing digesta viscosity in the gut (5). Consequently, the use of exogenous enzymes, such as NSP enzymes, has been suggested as a strategic approach to enhance nutrient utilization by breaking down fiber components, thereby improving digestibility and mitigating the potential negative effects associated with DDGS (6–10).

Broiler growth performance is significantly influenced by dietary composition across different feeding phases, as nutrient requirements vary from the starter to finisher phases. Assessing the impact of DDGS inclusion during these phases is essential for understanding its effects on body weight gain, feed efficiency, and overall productivity. Furthermore, immune responses are crucial for maintaining bird health and performance, with indicators such as immunoglobulin levels and antibody titers providing valuable insight into immune function. Intestinal histomorphology, particularly villus height and crypt depth, also serves as a key measure of gut health and nutrient absorption efficiency.

The present study investigated the effects of different levels of DDGS, with or without enzymes supplementation, on growth performance, immune responses, and intestinal histomorphology in broilers across feeding phases. The findings will contribute to optimizing DDGS utilization in poultry nutrition and support the maintenance of health and performance standards.

## 2 Materials and methods

All animal procedures and sample collection were conducted in accordance with national and institutional guidelines for the care and use of broilers and were approved by the Animal Ethics Committee of the Faculty of Veterinary Medicine, Menoufia University (Approval No. MN/VET/NUT/25/02/03/01).

### 2.1 Experimental diets

The experiment employed an experimental design involving eight distinct dietary treatments, conducted across two feeding phases: starter (0–14 days), and grower (15–28 days). Diets were formulated in accordance with the nutrient specifications for Avian 48 broilers. All experimental groups had similar raw material and nutrient contents, except for the DDGS and enzyme inclusion. The diets were provided in mash form. DDGS used in this experiment was imported from the United States, and its main nutrient composition as follows: Dry matter (DM), 88.42%; Crude protein (CP), 25%; Ether extract (EE), 8%; Crude fiber (CF), 7.9%; Ash, 4.79%; Nitrogen-free extract (NFE), 42.73%; and apparent metabolizable energy (AME), 2,525 Kcal/kg. Smart NSP enzymes® was the commercial feed additive used, obtained from Devenish Nutrition Ltd., 96 Duncrue Street, Belfast, BT3 9AR, Northern Ireland. This product contained a combination of 5 active enzymes (7,503 U/g xylanase, 2,500 U/g glucoamylase, 1,443 U/g β-glucanase, 375 U/g pectinase, 144 U/g cellulase) produced by advanced fermentation fungal strains. This enzyme provides an energy matrix of 1,050,000 Kcal/kg ME. The NSP enzymes were supplemented at a consistent rate of 100 mg·kg^−1^ in the relevant diets. Additionally, Smart phytase enzyme® (10,000 FTU/g phytase enzyme), derived from *Escherichia coli (E. coli)*, was also obtained from the commercial company Devenish Nutrition Ltd., Belfast, Northern Ireland. The composition of the treatment diets during the starter and grower phases is presented in Tables 1, 2, respectively.

**Table 1:** Composition and nutrient content of the starter experimental diets (as-fed basis).

Ingredients, g kg^−1^	Control	Control + Enz	5% DDGS	5% DDGS + Enz	10% DDGS	10% DDGS + Enz	20% DDGS	20% DDGS + Enz
Yellow corn, ground	552	576	525.7	543.2	498.5	512	432.2	446
Soybean meal (46%)	365	354	331	334	300	310	254	267
Corn gluten	16	20	24	19	30	20	33	20.5
DDGS	−	−	50	50	100	100	200	200
Vegetable oil	24.3	6.5	26	10.4	28	14	36	22
L-Lysine HCL (99%)	2.2	2.4	2.8	2.7	3.3	3.1	4.1	3.8
DL-Meth (99%)	2.4	2.5	2.4	2.4	2.2	2.3	2	2.1
Dicalcium phosphate	15	14.8	14.6	14.5	14.2	14.5	14.3	14.2
Limestone	13.05	13.65	13.45	13.65	13.75	13.95	14.35	14.25
Dietary supplement blend^a^	8.5	8.5	8.5	8.5	8.5	8.5	8.5	8.5
Mycotoxin binder^b^	1.5	1.5	1.5	1.5	1.5	1.5	1.5	1.5
NSP enzymes^c^	−	+	−	+	−	+	−	+
Phytase enzyme^d^	+	+	+	+	+	+	+	+
Calculated nutrients								
ME (kcal·kg^−1^ diet)^e^	3002.37	3003.3	3002.27	3002.13	3001.81	3001.2	3002.6	3001.14
Crude protein (%)^f^	22.86	22.85	22.85	22.84	22.83	22.80	22.84	22.80

a Dietary supplements and additives included: vitamin–mineral premix (3,000 mg·kg^−1^ inclusion, active vet Co.): each 1 kg of diet provided approximately: Vit. A (12,000 IU), Vit. D₃ (3,000 IU), Vit. E (7 mg), Vit. K₃ (5 mg), Vit. B₁ (5 mg), Vit. B₂ (2 mg), Vit. B₆ (6 mg), Vit. B₁₂ (30 μg), Vit. B₃ (30 mg), Vit. C (4.5 mg), choline chloride (10 mg), folic acid (3 mg), biotin (6 mg), copper (30 mg), iron (100 mg), zinc (1.8 mg), cobalt (3 mg), and magnesium (30 mg). Other dietary supplements: sodium bicarbonate (2000 mg·kg^−1^), common salt (2,500 mg·kg^−1^), and additional choline chloride (1,000 mg·kg^−1^).

b Mycotoxin binders include: Organic binder (EXTRA-TUKSINIL®, 500 mg·kg^−1^ inclusion; Hays Vet, China), containing mannanoligosaccharides (MOS, 100 g/kg), β-1,3-glucan (100 g/kg), formic acid (100 g/kg), lactic acid (100 g/kg), and propionic acid (100 g/kg). Inorganic binder (1,000 mg·kg^−1^ inclusion), consisting of hydrated sodium calcium aluminosilicate (HSCAS).

c NSP enzymes (Smart NSP enzymes®, 100 mg·kg^−1^ inclusion; Devenish Nutrition Ltd.): provided xylanase (7,503 U/g), glucoamylase (2,500 U/g), β-glucanase (1,443 U/g), pectinase (375 U/g), and cellulase (144 U/g); with an energy matrix of 1,050,000 kcal ME/kg.

d Phytase enzyme (Smart phytase®, 50 mg·kg^−1^ inclusion; Devenish Nutrition Ltd.): providing 10,000 FTU/g phytase activity derived from *E. coli*.

e ME values were calculated based on the analyzed chemical composition of each ingredient, using standard conversion factors Janssen (12).

f Crude proteins were calculated based on the analyzed chemical composition of each ingredient.

DDGS, dried distillers’ grains with solubles.

**Table 2:** Composition and nutrient content of the grower experimental diets (as-fed basis).

Ingredients, g kg^−1^	Control	Control + Enz	5% DDGS	5% DDGS + Enz	10% DDGS	10% DDGS + Enz	20% DDGS	20% DDGS + Enz
Yellow corn, ground	618	633.5	592.7	602.5	556	565.9	508.9	502
Soybean meal (46%)	295	298	258	275	244	260	165.7	213
Corn gluten	21.4	17	32	18	27	15	50	18.4
DDGS	−	−	50	50	100	100	200	200
Vegetable oil	28	13	29	16.3	34.6	21.1	35.8	28
L-Lysine HCL (99%)	2.1	2	2.8	2.4	3	2.5	4.4	3.2
DL-Meth (99%)	2	2	1.9	1.9	1.8	1.7	1.5	1.6
Dicalcium phosphate	12	12.5	11.8	12.05	11.7	11.7	11.5	11.7
Limestone	11.45	11.85	11.75	11.7	11.85	11.95	12.15	11.95
Dietary supplement blend^a^	8.5	8.5	8.5	8.5	8.5	8.5	8.5	8.5
Mycotoxin binder^b^	1.5	1.5	1.5	1.5	1.5	1.5	1.5	1.5
NSP enzymes^c^	−	+	−	+	−	+	−	+
Phytase enzyme^d^	+	+	+	+	+	+	+	+
Calculated nutrients								
ME (kcal·kg^−1^ diet)^e^	3102.75	3103.45	3102.04	3102.99	3102.84	3102.00	3102.1	3102.16
Crude protein (%)^f^	20.63	20.64	20.65	20.64	20.63	20.69	20.61	20.68

a Dietary supplements and additives included: vitamin–mineral premix (3,000 mg kg^−1^ inclusion; active Vet Co.): each 1 kg of diet provided approximately: Vit. A (12,000 IU), Vit. D₃ (3,000 IU), Vit. E (7 mg), Vit. K₃ (5 mg), Vit. B₁ (5 mg), Vit. B₂ (2 mg), Vit. B₆ (6 mg), Vit. B₁₂ (30 μg), Vit. B₃ (30 mg), Vit. C (4.5 mg), choline chloride (10 mg), folic acid (3 mg), biotin (6 mg), copper (30 mg), iron (100 mg), zinc (1.8 mg), cobalt (3 mg), and magnesium (30 mg). Other dietary supplements: sodium bicarbonate (2000 mg kg^−1^), common salt (2,500 mg kg^−1^), and additional choline chloride (1,000 mg kg^−1^).

b Mycotoxin binders include: organic binder (EXTRA-TUKSINIL®, 500 mg·kg^−1^ inclusion; Hays Vet, China), containing mannanoligosaccharides (MOS, 100 g/kg), β-1,3-glucan (100 g/kg), formic acid (100 g/kg), lactic acid (100 g/kg), and propionic acid (100 g/kg). Inorganic binder (1,000 mg·kg^−1^ inclusion), consisting of hydrated sodium calcium aluminosilicate (HSCAS).

c NSP enzymes (Smart NSP enzymes®, 100 mg kg^−1^ inclusion; Devenish Nutrition): provided xylanase (7,503 U/g), glucoamylase (2,500 U/g), β-glucanase (1,443 U/g), pectinase (375 U/g), and cellulase (144 U/g); with an energy matrix of 1,050,000 kcal ME/kg.

d Phytase enzyme (Smart phytase®, 50 mg·kg^−1^ inclusion; Devenish Nutrition): providing 10,000 FTU/g phytase activity derived from *E. coli*.

e ME values were calculated based on the analyzed chemical composition of each ingredient, using standard conversion factors Janssen (12).

f Crude proteins were calculated based on the analyzed chemical composition of each ingredient.

### 2.2 Broiler management

A total of 240 one-day-old broiler chicks (Avian 48; mean initial body weight 51.57 ± 4.6 g) were randomly allocated to 8 experimental groups in a completely randomized design, with 30 birds per group (three replicates of 10 birds each). The study was conducted over a 28-day feeding trial. Chicks were housed in floor pens with a litter-floor system, utilizing wood shavings as bedding material. All broilers were provided *ad libitum* access to experimental diets and water. Environmental conditions, including temperature, humidity, ventilation, and lighting, were monitored and adjusted according to the management guidelines for Avian 48 broilers. Broiler performance parameters, including body weight gain (BWG) and feed intake, were recorded weekly to evaluate growth performance and feed efficiency. Additionally, the feed conversion ratio (FCR) was calculated as the ratio of cumulative feed intake to cumulative body weight gain throughout the experimental period.

### 2.3 Chemical analysis

The components in the samples were determined using standard procedures of the Association of Official Analytical Chemists (11) for DM (934.01), CP (976.05), CF (962.09), and EE (920.39). ME values were calculated based on the analyzed chemical composition of each ingredient using the conversion factors described by Janssen (12) and expressed as a weighted sum according to their inclusion levels in the diet.

### 2.4 Vaccination program

Chicks were vaccinated at day 7 of age with EgyFlu®-ND7-3 in 1 (HARVAC, Harbin, China) via subcutaneous injection, providing inactivated protection against avian influenza virus (AIV; H5N8 and H9N2 subtypes) and RG-Newcastle disease virus (NDV; HB38 strain). Additionally, ocular administration of MEVAC LaSota+ H120® Elite (MEVAC, Egypt) was performed, delivering live lentogenic NDV (LaSota strain, 10^6^–10^7^ TCID₅₀) and attenuated infectious bronchitis virus (IBV; H120 strain, Massachusetts serotype, 10^3.5^–10^4.5^ EID₅₀). On day 14, booster vaccinations were administered via eye drops using RINNOVAC ELI-7®, a recombinant live NDV vaccine (VG/VG + F7 strains), and MEVAC IBD 818®, an intermediate-plus live vaccine against infectious bursal disease (IBD).

### 2.5 Blood sampling and biochemical analysis

Blood samples were collected weekly from three birds per replicate (*n* = 9 birds per treatment) at 7, 14, 21, and 28 days of age to assess hemagglutination inhibition (HI) titers against NDV and AIV. At the end of the trial, nine birds per group (three per replicate) were randomly selected for blood collection. Two types of samples were taken from the wing vein: one into plain tubes for serum separation (centrifuged at 3000 rpm for 10 min and stored at −18 °C), and the other into ethylene diamine tetra-acetic acid (EDTA) tubes for hematological analysis. Serum samples were analyzed using commercial diagnostic kits for total protein and its fractions (albumin and globulin), cholesterol, triglycerides, uric acid, creatinine, glutathione peroxidase (GPX), aspartate aminotransferase (AST), alanine aminotransferase (ALT), and immunoglobulin levels (IgA, IgM). Absorbance readings were obtained using a UV–visible spectrophotometer (UVS-85, Acculab, USA).

### 2.6 Immune organ evaluation

At the end of the experimental period, nine birds from each group (three per replicate) were randomly selected for immune organ evaluation. The Bursa of Fabricius, spleen, and thymus were carefully dissected and weighed to determine their absolute weights. Relative organ weights were calculated as (organ weight/live body weight) × 100 to account for individual variation.

### 2.7 Intestinal histomorphological analysis

**Figure 1:**
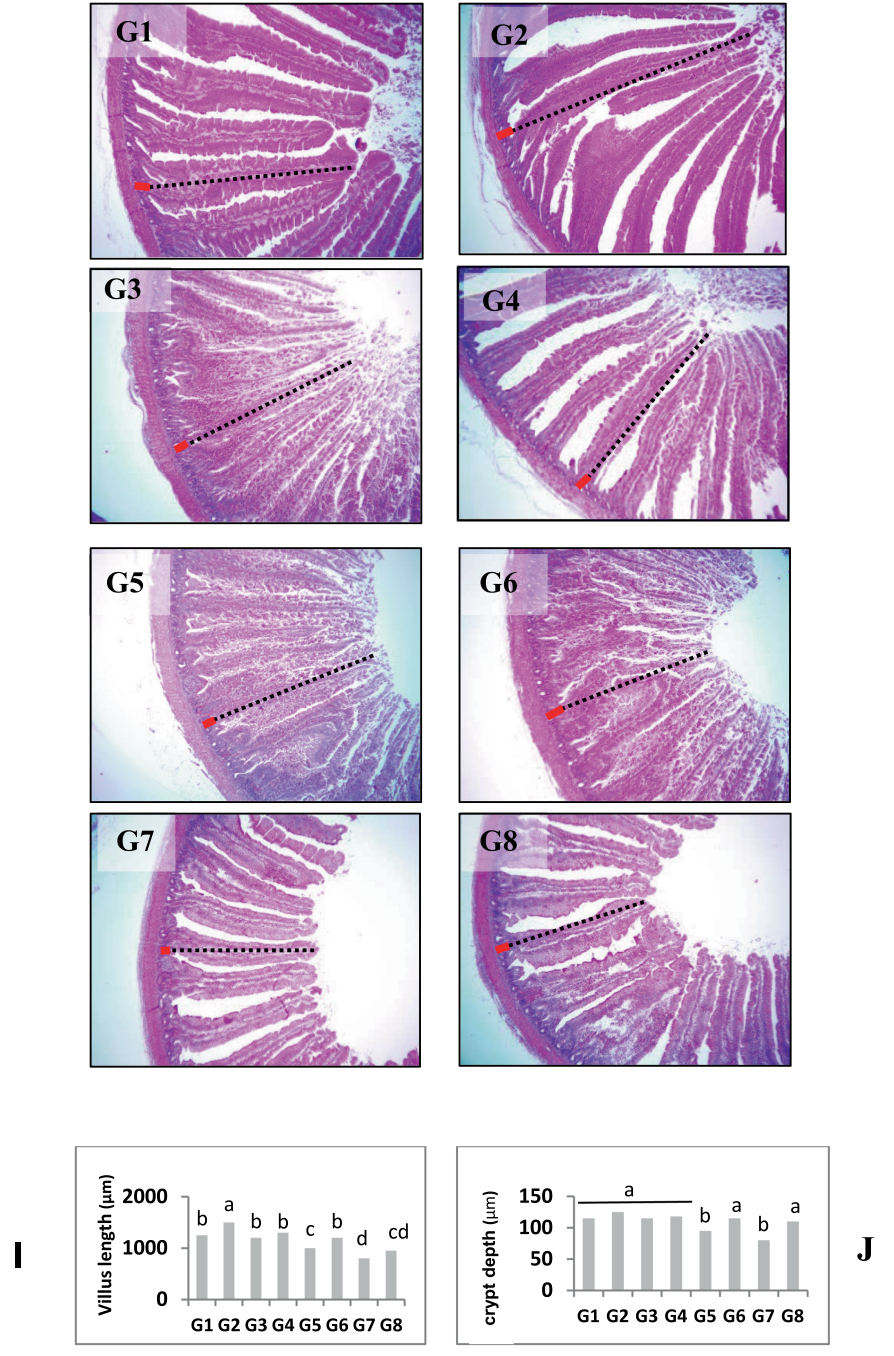
Histopathological examination of chicken jejunum samples from different groups. G1: control group, G2: basal diet plus NSP enzymes group, G3: 5% DDGS without NSP enzymes, G4: 5% DDGS plus NSP enzymes, G5: 10% DDGS without NSP enzymes, G6: 10% DDGS plus NSP enzymes, G7: 20% DDGS without enzymes, G8: 20% DDGS plus NSP enzymes, showing villus length (black dotted line), and crypt depth (red line). Scale bar = 500 μm. Image analysis of the studied groups for measuring villus length (I) and crypt depth (J). Bars with different letters are significantly different (*p* < 0.05).

At the end of the trial, jejunal samples were collected from two birds per replicate within each treatment. A 3-cm mid-jejunal segment (between the bile duct entry and Meckel’s diverticulum) was excised, rinsed with physiological saline to remove luminal contents, and fixed in 10% neutral-buffered formalin (100 mL 40% formaldehyde, 4 g monobasic phosphate, 6.5 g dibasic sodium phosphate, and 900 mL distilled water) for 18–24 h. Fixed tissues were dehydrated, embedded in paraffin, sectioned at 5 μm, and stained with hematoxylin and eosin (H&E) as described by Kim et al. (13). Slides were examined under a light microscope, and morphometric measurements (villus height and crypt depth) were performed using QuPath (version 0.4.3). Villus height (VH, μm) was measured from the tip of the villus to the villus–crypt junction, while crypt depth (CD, μm) was measured from the base of the villus to the bottom of the crypt. For each bird, ten well-oriented villi were evaluated in triplicate, and mean values were used for statistical analysis (see Figure 1).

### 2.8 Economic evaluation

An economic evaluation was conducted as a supportive and descriptive analysis to contextualize the biological performance results of broilers fed diets containing different levels of DDGS, with or without enzyme supplementation. Feed cost and return-related parameters were calculated based on actual feed intake, final live body weight, and prevailing market prices using standard economic calculations (14). Economic efficiency was expressed as the benefit–cost ratio (BCR) to provide practical insight into the potential economic implications of the dietary treatments.

### 2.9 Statistical analysis

Data were expressed as mean ± standard error (SE). All results were analyzed using two-way analysis of variance (ANOVA), and differences among means were evaluated using the least significant difference (LSD) *post hoc* test. Statistical analyses were performed using SPSS software (version 16.0; SPSS Inc., Chicago, IL, USA, 2001). A significance level of (*p* < 0.05) was considered statistically significant.

## 3 Results

### 3.1 Growth performance

Growth performance results in Table 3 showed that dietary DDGS levels and enzyme supplementation significantly (*p* < 0.05) affected BWG, FI, and FCR. During the starter period (0–14 days), the highest BWG was recorded in broilers fed the enzyme-supplemented basal diet, whereas the poorest growth occurred in birds receiving 20% DDGS without enzymes. Moderate DDGS levels (5–10%) combined with enzymes also improved BWG. Similar patterns were observed during the grower phase (15–28 days) and over the overall experimental period (0–28 days).

**Table 3:** Growth performance of broiler chickens offered varying dietary treatments (mean ± SE).

Group	Control	Control + Enz	5% DDGS	5% DDGS + Enz	10% DDGS	10% DDGS + Enz	20% DDGS	20% DDGS + Enz	*p*-values
Body weight gain (g)									
0–14 days	372.33 ± 2.27^d^	413.733 ± 2.18^a^	375.73 ± 1.54^d^	406.82 ± 1.13^b^	373.95 ± 1.32^d^	403.65 ± 0.57^b^	363.56 ± 1.74^e^	391.03 ± 1.02^c^	0.001***
15–28 days	844.58 ± 10.2^c^	930.26 ± 9.92^a^	861.71 ± 5.3^bc^	882.24 ± 16.2^b^	850.18 ± 7^c^	879.22 ± 2.78^b^	772.05 ± 15.9^e^	815.13 ± 5.57^d^	0.001***
Final BWG (0–28 days)	1216.7 ± 12.5^cd^	1342.8 ± 5.5^a^	1237.6 ± 7.9^c^	1289.1 ± 4.66^b^	1225.13 ± 9^cd^	1282.87 ± 9.9^b^	1135.6 ± 20.4^d^	1206.2 ± 7.9^cd^	0.002**
Feed consumption (g/chick)									
0–14 days	522.83 ± 7.16^d^	546 ± 7.50^cd^	548.67 ± 2.97^c^	557.62 ± 2.57^c^	551.07 ± 5.69^c^	560.57 ± 3.83^c^	624.57 ± 3.81^b^	643.67 ± 3.66^a^	0.004**
15–28 days	1620.5 ± 34.95^b^	1670.6 ± 12.41^ab^	1634.7 ± 25.4^ab^	1641.6 ± 24.1^ab^	1654.1 ± 25.44^ab^	1687.8 ± 31.9^a^	1670.7 ± 0.98^ab^	1676.4 ± 28.8^a^	0.021*
Total FI (0–28 days)	2144.33 ± 24^c^	2216.6 ± 32.78^bc^	2183.37 ± 40^c^	2198.2 ± 42.6^c^	2207.17 ± 9.7^bc^	2248.37 ± 56^ab^	2296.3 ± 24.8^ab^	2322.1 ± 38.7^a^	0.008**
Feed conversion ratio (FCR)									
0–14 days	1. 39 ± 0.012^bc^	1.31 ± 0.014^d^	1.45 ± 0.031^b^	1.37 ± 0.007^cd^	1.46 ± 0.01^b^	1.38 ± 0.032^bc^	1.71 ± 0.01^a^	1.64 ± 0.007^a^	0.001***
15–28 days	1.92 ± 0.029^c^	1.79 ± 0.014^d^	1.89 ± 0.027^cd^	1.85 ± 0.026^cd^	1.94 ± 0.031^c^	1.91 ± 0.035^c^	2.16 ± 0.009^a^	2.03 ± 0.02^b^	0.003**
Cumulative FCR (0–28 days)	1.76 ± 0.022^bc^	1.65 ± 0.042^d^	1.76 ± 0.015^bc^	1.71 ± 0.029^cd^	1.8 ± 0.068^b^	1.75 ± 0.051^bc^	2.02 ± 0.017^a^	1.93 ± 0.02^a^	0.012*

^a-d^Indicate significant differences between the dietary treatments.

Means in the same row with different superscripts differ significantly (*p* < 0.05). SE, standard error of the mean.

NS, Non-significant at (*p* > 0.05),*significant at (*p* < 0.05), **, significant at (*p* < 0.01), ***significant at (*p* < 0.001).

Feed intake was also significantly (*p* < 0.05) affected. In the early phase (0–14 days), diets with higher DDGS levels supplemented with enzymes were associated with higher feed intake, conversely, the lowest FI was observed in birds fed the control diet without DDGS. A similar trend was observed in the grower phase (15–28 days). Total FI (0–28 days) was higher in birds receiving enzyme-supplemented, high-DDGS diets. Feed utilization efficiency showed a consistent pattern. In the starter stage, birds receiving enzyme-supplemented diets recorded the most efficient FCR, while the poorest values were observed in diets containing high DDGS without enzymes. FCR progressively increased with DDGS inclusion across the entire period (0–28 days), though enzyme supplementation consistently provided a partial improvement.

### 3.2 Intestinal histomorphology

Histopathological examination and morphometric analysis of the intestinal samples revealed distinct structural variations among the dietary treatments. Birds receiving enzyme-supplemented diets, particularly those fed the basal diet or moderate DDGS inclusion levels (5–10%), exhibited well-developed intestinal villi characterized by increased villus height and comparatively reduced crypt depth. Conversely, birds fed the highest level of DDGS (20%) without enzyme inclusion showed marked reductions in villus height and increased crypt depth. Morphometric analysis confirmed these observations; as villus height (VH) was significantly lower (*p* < 0.05) in the high-DDGS group without enzymes. No significant differences (*p* > 0.05) were observed among the control, enzyme-supplemented, or moderate DDGS groups. Crypt depth (CD) remained statistically similar across most treatments, except in the high-DDGS group, which showed shallow crypts.

### 3.3 Physiological and immune status

#### 3.3.1 Serum biochemical parameters

Serum biochemical parameters presented in Table 4 showed significant differences (*p* < 0.05) among treatments in serum triglycerides and globulin levels. Conversely, no significant differences (*p* > 0.05) were found for cholesterol, total protein, albumin, A/G ratio, uric acid, creatinine, ALT, and AST levels. Specifically, groups receiving enzyme supplementation showed statistically higher levels in triglycerides and globulin compared to specific control groups. Furthermore, cholesterol levels were marginally reduced in DDGS-fed groups.

**Table 4:** Serum biochemical parameters of broilers in the different experimental groups (mean ± SE).

Parameter	Control	Control + Enz	5% DDGS	5% DDGS + Enz	10% DDGS	10% DDGS + Enz	20% DDGS	20% DDGS + Enz	*p*-values
Triglyceride mg/dL	164.29 ± 0^bc^	174.15 ± 1.4^a^	165.24 ± 3.3^bc^	170.07 ± 3.6^ab^	164.59 ± 2^bc^	170.1 ± 3.6^ab^	159.1 ± 0.95^c^	166.67 ± 2.38^abc^	0.019*
Cholesterol mg/dL	218.6 ± 14.9	226.95 ± 0.3	195.42 ± 4.18	201.89 ± 7.42	202.16 ± 1.7	203.77 ± 3.1	215.63 ± 15.8	220.76 ± 28.55	0.645 NS
total protein g/dL	2.82 ± 0.12	2.91 ± 0.29	2.86 ± 0.09	3.06 ± 0.09	2.89 ± 0.06	3.02 ± 0.03	2.8 ± 0.01	2.98 ± 0.11	0.999 NS
Albumin g/dL	1.5 ± 0.02	1.54 ± 0.03	1.45 ± 0.04	1.55 ± 0.03	1.45 ± 0.07	1.45 ± 0.02	1.45 ± 0.03	1.42 ± 0.04	0.994 NS
Globulin g/dl	1.35 ± 0.04^c^	1.37 ± 0.02 ^bc^	1.43 ± 0.02 ^abc^	1.5 ± 0.08 ^a^	1.39 ± 0.03 ^abc^	1.48 ± 0.03 ^ab^	1.32 ± 0.02 ^c^	1.4 ± 0.03 ^abc^	0.032*
A/G ratio	1.12 ± 0.05	1.12 ± 0.03	1.01 ± 0.03	0.99 ± 0.08	1.04 ± 0.04	0.98 ± 0.01	1.1 ± 0.02	1.01 ± 0.03	0.24 NS
Uric acid mg/dL	5.77 ± 0.12	5.92 ± 0.28	5.92 ± 0.18	5.9 ± 0.31	5.95 ± 0.26	5.83 ± 0.4	5.77 ± 0.4	5.83 ± 0.15	0.14 NS
Creatinine mg/dL	4.9 ± 0.39	5.32 ± 0.33	5.21 ± 0.65	5.36 ± 0.57	5.49 ± 0.12	5.18 ± 0.56	5.13 ± 0.36	5.2 ± 0.6	0.12 NS
ALT U/L	12.22 ± 1.01	12.8 ± 0.58	12.8 ± 1.16	12.8 ± 0.58	12.8 ± 1.16	12.8 ± 0.58	12.22 ± 1.01	12.8 ± 0.58	0.998 NS
AST U/L	267.14 ± 3.6	268.3 ± 3.5	267.72 ± 5.8	268.88 ± 2.02	263.65 ± 8.3	265.4 ± 4.4	267.72 ± 3.1	268.9 ± 8.79	0.997 NS

^a-d^Indicate significant differences between the dietary treatments.

Means in the same row with different superscripts differ significantly (*p* < 0.05).

NS, Non-significant at (*p* > 0.05), *significant at (*p* < 0.05), **, significant at (*p* < 0.01), ***significant at (*p* < 0.001).

#### 3.3.2 Immune response, antioxidant status, and hematological parameters

As presented in Table 5, the inclusion of DDGS, whether with or without enzyme supplementation, did not significantly (*p* > 0.05) influence the relative weights of the thymus and spleen. However, the relative weight of the Bursa of Fabricius was significantly influenced (*p* < 0.05) by the treatments. Specifically, Bursa weight was significantly (*p* < 0.05) higher in the enzyme-supplemented control group compared to the control group without enzymes, whereas other DDGS-fed groups with or without enzyme inclusion showed intermediate values.

**Table 5:** Effect of graded levels of dietary DDGS with or without enzyme supplementation on immune response, antioxidant status, and hematological parameters of broiler chickens (mean ± SE).

Parameter	Control	Control + Enz	5% DDGS	5% DDGS + Enz	10% DDGS	10% DDGS + Enz	20% DDGS	20% DDGS + Enz	*p*-values
Relative weight of lymphoid organs (%)									
Spleen %	0.1 ± 0.01	0.1 ± 0.007	0.11 ± 0.01	0.12 ± 0.01	0.11 ± 0.01	0.11 ± 0.01	0.11 ± 0.01	0.11 ± 0.01	0.616NS
Thymus %	0.31 ± 0.01	0.315 ± 0.01	0.31 ± 0.01	0.31 ± 0.014	0.32 ± 0.01	0.32 ± 0.01	0.315 ± 0.015	0.33 ± 0.01	0.824NS
Bursa of Fabricius %	0.11 ± 0.01^b^	0.142 ± 0.01^a^	0.12 ± 0.01^ab^	0.14 ± 0.02^ab^	0.12 ± 0.01^ab^	0.13 ± 0.01^ab^	0.11 ± 0.003^ab^	0.14 ± 0.01^ab^	0.016*
Immunoglobulins (mg/mL)									
IgA (mg/mL)	0.46 ± 0.03^c^	0.48 ± 0.05^bc^	0.5 ± 0.03^a^	0.51 ± 0.06 ^a^	0.49 ± 0.05 ^ab^	0.51 ± 0.08 ^a^	0.46 ± 0.03 ^c^	0.5 ± 0.04 ^a^	0.009**
IgM (mg/mL)	0.63 ± 0.05^c^	0.66 ± 0.06^ab^	0.66 ± 0.02^ab^	0.7 ± 0.02^a^	0.64 ± 0.01^bc^	0.69 ± 0.03^a^	0.61 ± 0.02^c^	0.69 ± 0.03^a^	0.005**
Antioxidative status (U/mL)									
GPX (U/mL)	259 ± 1.7	260 ± 1.8	264 ± 2.1	269 ± 1.33	268 ± 1.3	270 ± 2.33	260 ± 2.8	272 ± 1.32	0.197NS
Hematological parameters									
WBCs (×10^3^ /mm^3^)	20.37 ± 1.17^b^	21.24 ± 1.14^b^	27.05 ± 0.88^a^	28.05 ± 0.92^a^	27.58 ± 0.58^a^	28.69 ± 1.29^a^	21.52 ± 0.26^b^	26.94 ± 0.54^a^	0.02*
Lymphocytes %	69.92 ± 0.24^a^	68.50 ± 0.44^a^	65.24 ± 0.56^b^	62.19 ± 0.43^c^	65.65 ± 1.02^b^	62.68 ± 0.93^c^	65.16 ± 0.78^b^	65.16 ± 0.44^b^	0.003**
Heterophiles %	20.54 ± 0.36^c^	21.61 ± 0.55^c^	27.28 ± 0.53^b^	30.29 ± 0.45^a^	27.30 ± 0.74^b^	29.89 ± 0.94^a^	22.38 ± 0.57^c^	29.27 ± 0.57^a^	0.023*
H/L ratio	0.29 ± 0^c^	0.315 ± 0^c^	0.418 ± 0.01^b^	0.487 ± 0.01^a^	0.415 ± 0.02^b^	0.476 ± 0.02^a^	0.343 ± 0^c^	0.449 ± 0.13^ab^	0.011*

^a-d^Indicate significant differences between the dietary treatments.

Means in the same row with different superscripts differ significantly (*p* < 0.05). GPX, glutathione peroxidase; H/L ratio, heterophil to lymphocyte ratio.

NS, non-significant at (*p* > 0.05), *, significant at (*p* < 0.05), **, significant at (*p* < 0.01), ***, significant at (*p* < 0.001).

Regarding humoral immunity, serum IgA and IgM levels were significantly elevated (*p* < 0.05) in birds receiving enzyme-supplemented DDGS diets, particularly at 5–10% inclusion levels. Glutathione peroxidase (GPX) activity showed no significant differences (*p* > 0.05) among the groups. However, numerically higher values were observed in enzyme-supplemented DDGS treatments, especially at 10 and 20% inclusion levels. Hematological results revealed significantly higher total white blood cell counts (*p* < 0.05) in broilers fed diets containing 5 and 10% DDGS, particularly when combined with enzyme supplementation, compared to the control groups. In parallel, lymphocyte percentages were significantly reduced, while heterophil percentages and H/L ratios were markedly elevated in these groups.

#### 3.3.3 Serum antibody titers

As shown in Table 6, the ND titers varied significantly (*p* < 0.05) among groups on Day 7, Day 14, and Day 28, while no significant difference (*p* > 0.05) was observed on Day 21. Initially (Day 7), the highest titers were recorded in the control diets, but by Day 28, birds fed 10% DDGS showed significantly improved responses compared to the control group. Regarding H5N8, significant differences (*p* < 0.05) were detected across all measurement days. Birds fed 5% DDGS with enzymes had the significantly highest titers at Day 7, while the lowest titer at Day 28 was recorded in birds receiving 20% DDGS without enzyme supplementation. Overall, H5N8 titers declined over time. In contrast, H9N2 titers also showed significant differences (*p* < 0.05) across all days and generally increased over time. The highest levels were observed significantly at Day 28 in birds fed 20% DDGS.

**Table 6:** Effect of graded dietary levels of DDGS with or without enzyme supplementation on serum antibody titers against ND, H5N8, and H9N2 viruses in broiler chickens (mean ± SE).

Parameter		Control	Control + Enz	5% DDGS	5% DDGS + Enz	10% DDGS	10% DDGS + Enz	20% DDGS	20% DDGS + Enz	*p*-values
ND titers	Day 7	7.33 ± 0.24^a^	7 ± 0^ab^	6.66 ± 0.24^ab^	6.65 ± 0.47^ab^	5.66 ± 0.24^c^	5.66 ± 0.24^c^	6.33 ± 0.24^bc^	6.33 ± 0.24^bc^	0.001***
	Day 14	2.97 ± 0.41^ab^	2.97 ± 0.41^ab^	4.62 ± 0.63^a^	3.64 ± 0.47^ab^	2.57 ± 0.63^b^	2.57 ± 0.63^b^	2.97 ± 0.41^ab^	2.27 ± 0.48^b^	0.001***
	Day 21	5.25 ± 1.18	6.43 ± 0.57	5.33 ± 0.24	5.33 ± 0.24	5.33 ± 0.24	6.3 ± 0.62	5.94 ± 0.82	6.3 ± 0.62	0.69NS
	Day 28	4.95 ± 0.53^c^	5.99 ± 0.41^abc^	5.33 ± 0.24^c^	5.99 ± 0.41^abc^	6.66 ± 0.24^ab^	5.66 ± 0.24^bc^	6.99 ± 0.41^a^	6 ± 0^abc^	0.017*
H5N8 titers	Day 7	9.11 ± 1.06 ^ab^	8.33 ± 0.24 ^b^	8.31 ± 0.62^b^	10.99 ± 0.41^a^	9.31 ± 0.62^ab^	9.97 ± 0.82^ab^	10.33 ± 0.24^a^	9.99 ± 0.41^ab^	0.002**
	Day 14	5.66 ± 0.24 ^bc^	5.94 ± 0.82 ^bc^	6.64 ± 0.62^abc^	7.66 ± 0.24^a^	5.33 ± 0.24^c^	6.64 ± 0.62^abc^	7.33 ± 0.24^ab^	5.79 ± 0.76^bc^	0.024*
	Day 21	4 ± 0 ^ab^	3.28 ± 0.63 ^b^	4.66 ± 0.24^ab^	5 ± 0^a^	3.66 ± 0.24^ab^	3.61 ± 0.63^ab^	3.61 ± 0.63^ab^	4.65 ± 0.47^ab^	0.041*
	Day 28	2 ± 0 ^ab^	2.66 ± 0.24 ^ab^	2 ± 0^ab^	3.29 ± 0.47^a^	2.32 ± 0.24^ab^	3.14 ± 0.59^ab^	1.9 ± 0.71^b^	3.1 ± 0.5^ab^	0.038*
H9N2 titers	Day 7	7.66 ± 0.24 ^ab^	8.33 ± 0.24 ^a^	7.66 ± 0.24^ab^	7.64 ± 0.62^ab^	6.65 ± 0.47^b^	7.32 ± 0.47^ab^	7.99 ± 0.41^a^	8.33 ± 0.24^a^	0.014*
	Day 14	3.92 ± 0.71 ^bc^	3.61 ± 0.63 ^c^	5.65 ± 0.47^ab^	4.29 ± 0.63^abc^	3.9 ± 0.66^bc^	4.31 ± 0.69^abc^	5.99 ± 0.41^a^	4 ± 0^bc^	0.007**
	Day 21	6.58 ± 1.18 ^abc^	6.16 ± 1.44 ^abc^	5 ± 0^abc^	4.07 ± 1.28^c^	4.53 ± 1.04^bc^	6.96 ± 0.71^abc^	8.23 ± 1.25^a^	7.62 ± 0.85 ^ab^	0.019*
	Day 28	9.75 ± 0.38^ab^	10 ± 0^ab^	9.63 ± 0.37^b^	9.31 ± 0.62^b^	9.66 ± 0.24^b^	9.32 ± 0.47^b^	10.99 ± 0.41^a^	10.33 ± 0.24^ab^	0.046*

^a-d^Indicate significant differences between the dietary treatments.

Means in the same row with different superscripts differ significantly (*p* < 0.05). ND, Newcastle disease; H5N8, H9N2, Avian influenza virus subtypes.

NS, non-significant at (*p* > 0.05), *, significant at (*p* < 0.05), **, significant at (*p* < 0.01), ***, significant at (*p* < 0.001).

### 3.4 Economical evaluation

The economic-related parameters of broilers fed the experimental diets are summarized in Table 7. Statistically significant differences (*p* < 0.01) were detected among the experimental groups for the calculated economic indicators.

**Table 7:** Productive, economic parameters and economic efficiency measures among different groups (value/bird).

Parameter	Control	Control + Enz	5%DDGS	5% DDGS + Enz	10% DDGS	10% DDGS + Enz	20% DDGS	20% DDGS + Enz	*p*-values
Total feed cost	67.80 ± 7.80^b^	68.15 ± 8.1^b^	68.11 ± 8.1^b^	65.96 ± 6.99^b^	69.39 ± 9.1^b^	67.98 ± 9.8^b^	72.16 ± 7.11^a^	71.23 ± 7.12^a^	0.003**
Total costs	142.8 ± 8.12^b^	143.15 ± 10.1^b^	143.11 ± 11.1^b^	140.96 ± 10.14^c^	144.39 ± 13.1^b^	142.98 ± 12.1^b^	147.16 ± 11.1^a^	146.23 ± 14.1^a^	0.009**
Value of final bird	146.49 ± 4.90^d^	167.73 ± 7.33^a^	150.63 ± 6.33^c^	157.89 ± 7.80^b^	148.67 ± 4.85^d^	156.59 ± 5.90^b^	132.09 ± 3.29^e^	146.54 ± 5.44^d^	0.001***
Total return	156.49 ± 16.4^d^	177.73 ± 17.37^a^	160.63 ± 16.4^c^	167.89 ± 18.19^b^	158.67 ± 17.2^d^	166.59 ± 15.2^b^	142.09 ± 14.1^e^	156.54 ± 14.2^d^	0.001***
Net profit	13.69 ± 2.69^e^	34.58 ± 5.8^a^	17.52 ± 5.22^d^	26.93 ± 3.14^b^	14.28 ± 4.1^e^	23.61 ± 3.1^c^	−5.07 ± 1.15^g^	10.31 ± 2.58^f^	0.001***
Feed costs/total returns	43.33 ± 3.34^c^	38.34 ± 4.12 ^e^	42.40 ± 4.14 ^c^	39.29 ± 3.12 ^e^	43.73 ± 4.15 ^c^	40.81 ± 4.18 ^d^	50.78 ± 5.17 ^a^	45.50 ± 5.17^b^	0.002**
Net profit/total costs	9.59 ± 1.19^e^	24.16 ± 4.15^a^	12.24 ± 4.12^d^	19.10 ± 3.14^b^	9.89 ± 4.15^e^	16.51 ± 5.14^c^	−3.45- ± 1.14^g^	7.05 ± 2.15^f^	0.001**
Benefit/cost ratio	109.59 ± 5.9^e^	124.16 ± 10.1^a^	112.24 ± 12.1^d^	119.10 ± 10.14^b^	109.89 ± 10.2^e^	116.51 ± 11.1^c^	96.55 ± 5.15^g^	107.05 ± 10.2^f^	0.001***

^a-d^Indicate significant differences between the dietary treatments.

Means within the same row of different litters are significantly different at (*P* < 0.01).

NS, non-significant at (*p* > 0.05), *significant at (*p* < 0.05), **significant at (*p* < 0.01), ***significant at (*p* < 0.001).

## 4 Discussion

The results indicated that enzyme supplementation significantly improved BWG and FCR, particularly in the basal diet, highlighting the positive role of enzyme inclusion, especially non-starch polysaccharide-degrading enzymes, in enhancing early nutrient digestibility and utilization (6–10). The observation that moderate levels of DDGS (5–10%) combined with enzymes also showed improved BWG is consistent with earlier findings (15), whereas birds fed the highest DDGS level (20%) without enzymatic support displayed the poorest growth, is consistent with previous studies that report reduced nutrient availability associated with high DDGS inclusion in the absence of enzymes (16).

The poorest performance observed in the high DDGS group without enzymes further emphasizes the challenges posed by high fiber and associated anti-nutritional compounds. Although enzyme inclusion in high-DDGS diets led to numerical improvements, the gains did not match those achieved with enzyme-supplemented diets free from high DDGS. These findings highlight the partial efficacy of enzymes in mitigating the negative effects of high DDGS inclusion, in line with the observations of previous studies (17). By the end of the period (0–28 days), the cumulative effect demonstrated that enzyme-supplemented groups at low to moderate DDGS levels maintained higher gains, while birds on unsupplemented high-DDGS diets showed the lowest BWG (18).

The significant effect on feed intake in the early phase (*p* < 0.05), where diets with higher DDGS levels plus enzymes stimulated greater feed consumption, is possibly due to compensatory feeding behavior and the higher fiber content of DDGS (4). Birds fed enzyme-supplemented diets consumed more feed than the control group (19, 20). These findings are consistent with reports indicating that early-phase FI increases with moderate DDGS inclusion but may decline with extended feeding and higher levels (21). The higher total FI (0–28 days) in enzyme-supplemented, high-DDGS diets further emphasizes the role of enzymes in enhancing palatability and digestion efficiency in fiber-rich diets.

The consistent pattern of FCR across phases, where DDGS inclusion progressively increased FCR, reflects the negative impact of dietary fiber and associated anti-nutritional factors on nutrient utilization. Enzyme supplementation consistently improved FCR across all inclusion levels, but the improvement was only partial and did not completely restore feed efficiency to the enzyme-supplemented control. These findings indicate that DDGS inclusion reduces nutrient digestibility and feed efficiency, while enzyme supplementation can alleviate, but not eliminate, the adverse effects. These findings align with the pattern documented previously (22).

The observation that birds receiving enzyme-supplemented diets exhibited well-developed intestinal villi with increased height and reduced crypt depth indicates enhanced absorptive capacity and improved mucosal integrity, supporting the role of enzymes in promoting gut health. Similar improvements in intestinal morphology with enzyme supplementation have been observed in previous studies (23), conversely, the marked reductions in villus height and increased crypt depth observed in birds fed the highest level of DDGS without enzyme inclusion suggest compromised intestinal function and nutrient absorption. These alterations are in line with reports indicating that DDGS levels above 15% may adversely affect intestinal morphology (24).

The morphometric analysis, confirming that the high-DDGS group without enzymes had significantly lower villus height and shallow crypts (*p* < 0.05), reflects the negative impact of high fiber and anti-nutritional factors. The shallow crypts suggest reduced epithelial turnover in response to impaired villus growth, which is a key indicator of poor gut health and reduced capability for nutrient absorption.

The finding that most serum biochemical parameters (cholesterol, total protein, albumin, A/G ratio, uric acid, creatinine, ALT, and AST) remained unaffected (*p* > 0.05) partially agrees with earlier observations indicating no significant alterations in plasma biochemical indices with dietary DDGS inclusion up to 20% in poultry (25). This suggests that incorporating DDGS, with or without NSP enzymes, does not adversely affect overall broiler metabolism, and did not compromise liver or kidney function. However, the observation of statistically higher levels in triglycerides and globulin (*p* < 0.05) in enzyme-supplemented groups compared to specific control groups indicates enhanced nutrient utilization or metabolic activity related to these components. Similar trends were described in studies involving DDGS inclusion up to 16% (26). The marginal reduction in cholesterol levels observed in DDGS-fed groups is possibly due to the cholesterol-lowering effect of dietary fiber inherent in DDGS, as previously noted (27).

The finding that Bursa of Fabricius weight was significantly influenced (*p* < 0.05) by the treatments, contrasting with the thymus and spleen, partially supports previous findings (27, 28), which reported no negative impact of DDGS (up to 15–20%) on immune development. The significantly higher bursa weight observed in the enzyme-supplemented control group, with other DDGS-fed groups showing intermediate values, suggests enhanced immune tissue development without compromising physiological function. The observation that IgA and IgM levels were significantly elevated (*p* < 0.05) in enzyme-supplemented DDGS diets, reflecting enhanced immune responsiveness, aligns with findings attributing improved antibody levels to the prebiotic effects of yeast components present in DDGS (29, 30). While Glutathione peroxidase (GPX) activity showed no significant differences (*p* > 0.05), the numerically higher values observed in enzyme-supplemented DDGS treatments support previous findings that DDGS may enhance antioxidant enzyme activity through its polyunsaturated fatty acid content and sulfur-containing amino acids, as indicated (31, 32). The revealed hematological shifts, including significantly higher WBC counts and H/L ratios in DDGS-enzyme groups, may reflect physiological immune stimulation or mild systemic stress, potentially driven by increased metabolic demand or the presence of DDGS fermentation by-products. This interpretation aligns with the findings that elevated WBCs and H/L ratios are reliable indicators of immune activation and stress responses in poultry, as previously documented (33).

The significant variation in ND titers across different days, with 10% DDGS showing significantly improved responses compared to the control group by Day 28, suggests potential long-term immunomodulatory effects of the diet. Regarding H5N8, the overall decline in titers over time could be attributed to environmental exposure to circulating H5N8 viral particles, natural waning of immunity, and reduced vaccine efficacy over time, in agreement with earlier observations (34). In contrast, the significant increase in H9N2 titers over time, with the highest levels observed significantly at Day 28 in birds fed 20% DDGS, indicates an enhanced late-phase immune response to this antigen in the higher DDGS inclusion groups.

Economic efficiency was evaluated as a secondary outcome derived from growth performance and feed utilization parameters. The observed variation in economic indicators, where the highest total cost was observed in broilers fed the 20% DDGS diet without enzymes, whereas the lowest cost was recorded in birds receiving the 5% DDGS diet supplemented with enzymes, a trend that is consistent with the findings of reports indicating a reduction in feed cost per kilogram of gain when moderate levels of DDGS were combined with enzyme supplementation (35). Although total costs were relatively comparable among most treatments, broilers fed the basal diet with enzyme supplementation and those receiving 5% DDGS plus enzymes achieved the highest final bird price and total return (*p* < 0.01), reflecting superior growth performance and feed utilization. Consequently, these groups also recorded the highest net profits, followed by the 10% DDGS with enzyme supplementation group. In contrast, the negative economic returns observed in broilers fed 20% DDGS without enzymes highlight the economic penalty associated with reduced nutrient availability and impaired performance, likely due to increased levels of anti-nutritional factors at high DDGS inclusion. These findings are consistent with previous reports (15), confirming that enzyme supplementation is a critical strategy for enhancing economic efficiency and maximizing profitability when incorporating DDGS into broiler diets.

## 5 Conclusion

Dietary inclusion of DDGS at moderate levels (5–10%) throughout the starter, and grower phases, when combined with NSP enzyme supplementation, improved broiler performance, immune response, intestinal morphology, and overall economic efficiency without negative effects on metabolism. Although the control diet with enzyme supplementation showed the best overall performance.

## Data Availability

The original contributions presented in the study are included in the article/supplementary material, further inquiries can be directed to the corresponding authors.

## References

[1] Bao G Wei H He D Wang D Ding B Zhang J . Dietary supplementation with corn distillers’ dried grains with solubles improves egg quality in Haidong chickens by promoting beneficial gut microbiota and modulating serum biochemical profiles. Front Microbiol. (2025) 16:1709407. doi: 10.3389/fmicb.2025.1709407, 41395495 PMC12698570

[2] Biofuels International. Global biofuel demand to grow 20% in next five years. Biofuels Int. (2023). Available online at: https://biofuels-news.com/news/global-biofuel-demand-to-grow-20-in-next-five-years/ (accessed October 11, 2024)

[3] Świa,tkiewicz S Koreleski J. Effect of dietary level of maize- and rye distiller dried grains with solubles on nutrient utilization and digesta viscosity in laying hens. J Anim Feed Sci. (2007) 16:668–77. doi: 10.22358/jafs/66824/2007

[4] Batal AB Parsons CM. Effects of age on nutrient digestibility in chicks fed different diets. Poult Sci. (2002) 81:400–7. doi: 10.1093/ps/81.3.400, 11902418

[5] Ward AT Marquardt RR. The effect of saturation, chain length of pure triglycerides, and age of bird on the utilization of rye diets. Poult Sci. (1983) 62:1054–62. doi: 10.3382/ps.0621054, 6878135

[6] Yuan L Wang M Zhang X Wang Z. Effects of protease and non-starch polysaccharide enzyme on performance, digestive function, activity and gene expression of endogenous enzyme of broilers. PLoS One. (2017) 12:1–13. doi: 10.1371/journal.pone.0173941, 28323908 PMC5360255

[7] Kumar R Tiwari RK Kumari A Shahi B Singh KM Saha SK . Effect of supplementation of non-starch polysaccharide cocktail enzyme on performance in broiler. J AgriSearch. (2019) 6:95–100.

[8] Yaghobfar A Kalantar M. Effect of non–starch polysaccharide (NSP) of wheat and barley supplemented with exogenous enzyme blend on growth performance, gut microbial, pancreatic enzyme activities, expression of glucose transporter (SGLT1) and MUCIN producer (MUC2) genes of broiler. Rev Bras Cienc Avic / Brazilian J Poult Sci. (2017) 19:629–38. doi: 10.1590/1806-9061-2016-0441

[9] Swiatkiewicz S Arczewska-Wlosek A Jozefiak D. Feed enzymes, probiotic, or chitosan can improve the nutritional efficacy of broiler chicken diets containing a high level of distillers dried grains with solubles. Livest Sci. (2014) 163:110–9. doi: 10.1016/j.livsci.2014.03.001

[10] Alam MJ Howlider MAR Pramanik MAH Haque MA. Effect of exogenous enzyme in diet on broiler performance. Int J Poult Sci. (2003) 2:168–73. doi: 10.3923/ijps.2003.168.173

[11] Association of Official Analytical Chemists. Official methods of analysis of AOAC international. 17th ed. Gaithersburg, MD: AOAC International (2003).

[12] Janssen WMMA. European table of energy values for poultry feedstuffs: Substitution method. 3rd ed. Beekbergen, The Netherlands: Spelderholt Centre for Poultry Research and Information Services (1989).

[13] Kim MJ Ingale SL Hosseindoust A Choi YH Kim KY Chae BJ. Synergistic effect of exogenous multi-enzyme and phytase on growth performance, nutrients digestibility, blood metabolites, intestinal microflora and morphology in broilers fed corn-wheat-soybean meal diets. Anim Biosci. (2021) 34:1365–74. doi: 10.5713/ab.20.0663, 33561925 PMC8255893

[14] Adaszyńska-Skwirzyńska M Konieczka P Bucław M Majewska D Pietruszka A Zych S . Analysis of the production and economic indicators of broiler chicken rearing in 2020–2023: a case study of a polish farm. Agri. (2025) 15:1–11. doi: 10.3390/agriculture15020139

[15] Jin D Tugiyanti E Rimbawanto EA Rosidi R Widiyastuti T Susanto A . Effects of high-level dietary distillers dried grains with solubles supplemented with multienzymes on growth performance, nutrient utilization, intestinal morphology, and pellet quality in broiler chickens. Vet World. (2024) 17:1943–54. doi: 10.14202/vetworld.2024.1943-1954, 39328431 PMC11422655

[16] Youssef AW Abd El-Azeem NA El-Daly EF El-Monairy MM. The impact of feeding graded levels of distillers dried grains with solubles (DDGS) on broiler performance, hematological and histological parameters. Asian J Poult Sci. (2013) 7:41–54. doi: 10.3923/AJPSAJ.2013.41.54

[17] Attia YA Al-Khalaifah HS Alqhtani AH Abd El-Hamid HS Alyileili SR El-Hamid AE-HEA . The impact of multi-enzyme fortification on growth performance, intestinal morphology, nutrient digestibility, and meat quality of broiler chickens fed a standard or low-density diet. Front Vet Sci. (2022) 9:1012462. doi: 10.3389/fvets.2022.1012462, 36504838 PMC9731804

[18] Shams-eldin AM Ahmed AS Elbestawy AR Ahmed HA. Effect of using distillers dried grains with solubles of corn as an unconventional feedstuff on broiler performance. Menoufia Vet Med J. (2025) 1:1–12. doi: 10.21608/vmmj.2025.359302.1027

[19] Amal MH Ka M Mukhtar A. Comparative study to evaluate nutritive value of maize and sorghum (feteria) with or without commercial enzyme (xylem) in broiler diets. J Glob Biosci. (2015) 4:2296–303.

[20] Habib A Mohamed A Eltrifi A Abu-Shullukh E Abubaker A. Effect of feed supplemented with Xylam enzyme on performance, carcass characteristics and meat quality of broiler chicks. J Appl Vet Sci. (2016) 1:15–20. doi: 10.21608/javs.2016.61822

[21] Liu N Ru YJ Tang DF Xu TS Partridge GG. Effects of corn distillers dried grains with solubles and xylanase on growth performance and digestibility of diet components in broilers. Anim Feed Sci Technol. (2011) 163:260–6. doi: 10.1016/j.anifeedsci.2010.11.004

[22] Foltyn M Rada V Lichovníková M Dracková E. Effect of corn DDGS on broilers performance and meat quality. Acta Univ Agric Silvic Mendel Brun. (2013) 61:59–64. doi: 10.11118/actaun201361010059

[23] Gabriela CDP Lee A Bortoluzzi C Rohloff Junior N Farnell YZ Pilla R . Distillers dried grains with soluble and enzyme inclusion in the diet effects broilers performance, intestinal health, and microbiota composition. Poult Sci. (2023) 102:102981. doi: 10.1016/j.psj.2023.102981, 37742451 PMC10523001

[24] Min YN Li HL Li L Niu ZY Wang JJ Liu SK . Effects of dietary distillers dried grains with solubles (DDGS) concentrations on intestinal morphology of broiler chicken. J Anim Vet Adv. (2013) 12:6–9.

[25] Wickramasuriya SS MacElline SP Kim E Cho HM Shin TK Yi YJ . Physiological impact on layer chickens fed corn distiller’s dried grains with solubles naturally contaminated with deoxynivalenol. Asian Australas J Anim Sci. (2020) 33:313–22. doi: 10.5713/ajas.19.0199, 31480205 PMC6946975

[26] Damasceno JL Rocha CS Eyng C Broch J Savaris VDL Wachholz L . Corn distillers’ dried grains with solubles to feed broiler chickens from 22 to 42 D of age. J Appl Poult Res. (2020) 29:573–83. doi: 10.1016/j.japr.2020.03.004

[27] Elbaz AM Gad GG Thabet HA. Effect of feeding corn distillers dried grains with solubles. Egypt J Nutr Feed. (2022) 25:85–94. doi: 10.21608/ejnf.2022.236573

[28] Min YN Li LL Liu SK Zhang J Gao YP Liu FZ. Effects of dietary distillers dried grains with solubles (DDGS) on growth performance, oxidative stress, and immune function in broiler chickens. J Appl Poult Res. (2015) 24:23–9. doi: 10.3382/japr/pfv002

[29] Fathi MM Ebeid TA Al-Homidan I Soliman NK Abou-Emera OK. Influence of probiotic supplementation on immune response in broilers raised under hot climate. Br Poult Sci. (2017) 58:512–6. doi: 10.1080/00071668.2017.1332405, 28521530

[30] Lim C Yildirim-Aksoy M Klesius PH. Growth response and resistance to *Edwardsiella ictaluri* of channel catfish, *Ictalurus punctatus*, fed diets containing distiller’s dried grains with solubles. J World Aquac Soc. (2009) 40:182–93. doi: 10.1111/j.1749-7345.2009.00241.x

[31] Ruan D Fouad AM Fan QL Chen W Xia WG Wang S . Effects of corn dried distillers’ grains with solubles on performance, egg quality, yolk fatty acid composition and oxidative status in laying ducks. Poult Sci. (2018) 97:568–77. doi: 10.3382/ps/pex331, 29211867

[32] Heincinger M Balogh K Fébel H Erdélyi M Mézes M. Effect of diets with different inclusion levels of distillers dried grain with solubles combined with lysine and methionine supplementation on the lipid peroxidation and glutathione status of chickens. Acta Vet Hung. (2011) 59:195–204. doi: 10.1556/AVet.2011.005, 21665573

[33] Ribeiro LRR Sans EC d O Santos RM Taconelli CA de Farias R Molento CFM. Will the white blood cells tell? A potential novel tool to assess broiler chicken welfare. Front Vet Sci. (2024) 11:1–16. doi: 10.3389/fvets.2024.1384802, 39015105 PMC11250086

[34] Doyon-Plourde P Przepiorkowski J Young K Zhao L Sinilaite A. Intraseasonal waning immunity of seasonal influenza vaccine—a systematic review and meta-analysis. Vaccine. (2023) 41:4462–71. doi: 10.1016/j.vaccine.2023.06.038, 37331840

[35] Choi HS Lee HL Shin MH Jo C Lee SK Lee BD. Nutritive and economic values of corn distiller’s dried grains with solubles in broiler diets. Asian Australas J Anim Sci. (2008) 21:414–9. doi: 10.5713/ajas.2008.70067

